# Integrative multi-omics reveals two biologically distinct groups of pilocytic astrocytoma

**DOI:** 10.1007/s00401-023-02626-5

**Published:** 2023-09-01

**Authors:** Daniel Picard, Jörg Felsberg, Maike Langini, Paweł Stachura, Nan Qin, Jadranka Macas, Yvonne Reiss, Jasmin Bartl, Florian Selt, Romain Sigaud, Frauke-D. Meyer, Anja Stefanski, Kai Stühler, Lucia Roque, Rafael Roque, Aleksandra A. Pandyra, Triantafyllia Brozou, Christiane Knobbe-Thomsen, Karl H. Plate, Alexander Roesch, Till Milde, Guido Reifenberger, Gabriel Leprivier, Claudia C. Faria, Marc Remke

**Affiliations:** 1 Department of Pediatric Oncology, Hematology, and Clinical Immunology, Medical Faculty, and University Hospital Düsseldorf, Heinrich Heine University, Düsseldorf, Germany; 2 German Cancer Consortium (DKTK), Partner site Essen/Düsseldorf, Düsseldorf, Germany; 3 Institute of Neuropathology, Medical Faculty, and University Hospital Düsseldorf, Heinrich Heine University, Düsseldorf, Germany; 4 Molecular Proteomics Laboratory, Biological and Medical Research Center (BMFZ), Heinrich Heine University Düsseldorf, Düsseldorf, Germany; 5 Institute for Molecular Medicine I, Heinrich Heine University Medical Faculty, Düsseldorf, Germany; 6 Institute for Molecular Medicine II, Heinrich Heine University Medical Faculty, Düsseldorf, Germany; 7 Institute of Neurology (Edinger Institute), University Hospital Frankfurt, Frankfurt am Main, Germany; 8 German Cancer Consortium (DKTK), Partner site Frankfurt/Mainz, Frankfurt, Germany; 9 Frankfurt Cancer Institute, Frankfurt, Germany; 10 Hopp Children’s Cancer Center Heidelberg (KiTZ), Heidelberg, Germany; 11 Clinical Cooperation Unit Pediatric Oncology, German Cancer Research Center (DKFZ) and German Cancer Consortium (DKTK), Heidelberg, Germany; 12 KiTZ Clinical Trial Unit (ZIPO), Department of Pediatric Hematology and Oncology, University Hospital Heidelberg, Heidelberg, Germany; 13 Portuguese Cancer Institute, Unidade de Investigação em Patobiologia Molecular (UIPM), IPOLFG, Lisbon, Portugal; 14 Laboratory of Neuropathology, Neurology Department, Hospital de Santa Maria, Centro Hospitalar Universitário Lisboa Norte (CHULN), Lisbon, Portugal; 15 Institute of Clinical Chemistry and Clinical Pharmacology, University Hospital Bonn, Bonn, Germany; 16 German Center for Infection Research (DZIF), Partner Site Bonn-Cologne, Bonn, Germany; 17 Department of Dermatology, University Hospital Essen, West German Cancer Center, University Duisburg-Essen, Essen, Germany; 18 Center for Medical Biotechnology (ZMB), University of Duisburg-Essen, Essen, Germany; 19 National Center for Tumor Diseases (NCT), Heidelberg, Germany; 20 Faculdade de Medicina, Instituto de Medicina Molecular João Lobo Antunes, da Universidade de Lisboa, Lisbon, Portugal; 21 Department of Neurosurgery, Hospital de Santa Maria, Centro Hospitalar Universitário Lisboa Norte (CHULN), Lisbon, Portugal

**Keywords:** Pilocytic astrocytoma, Intertumoral heterogeneity, Integrative multi-omics

## Abstract

Pilocytic astrocytoma (PA), the most common pediatric brain tumor, is driven by aberrant mitogen-activated protein kinase signaling most commonly caused by *BRAF* gene fusions or activating mutations. While 5-year overall survival rates exceed 95%, tumor recurrence or progression constitutes a major clinical challenge in incompletely resected tumors. Here, we used similarity network fusion (SNF) analysis in an integrative multi-omics approach employing RNA transcriptomic and mass spectrometry-based proteomic profiling to molecularly characterize PA tissue samples from 62 patients. Thereby, we uncovered that PAs segregated into two molecularly distinct groups, namely, Group 1 and Group 2, which were validated in three non-overlapping cohorts. Patients with Group 1 tumors were significantly younger and showed worse progression-free survival compared to patients with group 2 tumors. Ingenuity pathways analysis (IPA) and gene set enrichment analysis (GSEA) revealed that Group 1 tumors were enriched for immune response pathways, such as interferon signaling, while Group 2 tumors showed enrichment for action potential and neurotransmitter signaling pathways. Analysis of immune cell-related gene signatures showed an enrichment of infiltrating T Cells in Group 1 versus Group 2 tumors. Taken together, integrative multi-omics of PA identified biologically distinct and prognostically relevant tumor groups that may improve risk stratification of this single pathway driven tumor type.

**Supplementary Information:**

The online version contains supplementary material available at 10.1007/s00401-023-02626-5.

## Introduction

Pilocytic astrocytomas (PAs) are the most common primary brain tumors in children [29]. The majority of tumors develop in the cerebellum, followed by less common locations in other midline structures, such as the optic nerve and chiasm, the hypothalamus and the spinal cord, and by locations in the cerebral cortex [40]. PAs are typically slowly growing, and, if well-circumscribed, can be successfully treated by surgery [28], with 5-year overall survival rates exceeding 95%. Subtotally resected or unresectable tumors due to tumor location, e.g., tumors located in the optic tract and hypothalamus, tend to recur and may require adjuvant therapy by local irradiation or systemic chemotherapy [9]. However, approximately 55% of these tumors progress following current standard of care treatment, and novel treatment strategies are thus urgently needed [24].

Concerning pathogenesis, PAs are considered as a single pathway disease driven by genetic alterations of genes encoding members of the mitogen-activated protein kinase (MAPK) signaling cascade, with the majority of tumors, in particular among the cerebellar PAs, carrying *KIAA1549::* B-Raf proto-oncogene, serine/threonine kinase (*BRAF*) fusions that lead to aberrant MAPK pathway activation [16]. Less common genetic alterations in PAs include activating *BRAF* codon 600 mutations, inactivating neurofibromin 1 (*NF1*) mutations or rarely alterations in the fibroblast growth factor receptor 1/2 (*FGFR1/2*), protein tyrosine phosphatase non-receptor type 11 (*PTPN11*) or neurotrophic receptor tyrosine kinase (*NTRK*) genes [16]. Based on these molecular findings, individual case observations as well as early clinical trials have been focusing on pharmacological inhibition of MAPK signaling using BRAF and/or MAPK/ERK kinase (MEK) inhibitors as a molecularly guided strategy for targeted therapy (for review see Ruda et al*.* [37]). Thus far, available data suggest that this targeted strategy may improve outcome of patients with recurrent, refractory or progressive disease. For example, several studies on pediatric patients with *BRAF*- or *NF1*-altered progressive or recurrent low-grade gliomas (LGG), including PAs, reported on promising clinical signals for targeted treatments with BRAF or MEK inhibitors when compared to control patients receiving the standard-of-care [4, 10, 13]. It is also interesting to note that patients who stopped treatment had rapid tumor re-growth [27].

Several molecular profiling studies have been conducted that aimed to stratify PA patients based on large-scale gene expression and/or DNA methylation profiling data [1, 18, 20, 38, 48]. In particular, studies have analyzed epigenetic data to define molecular groups [20, 38]. However, upon clustering of these cohorts, the tumors largely separated according to their anatomical location. Thus, there is no current consensus on subgrouping of PAs based on molecular markers or signatures, indicating a low degree of biological heterogeneity among these tumors. Notably, however, previous molecular profiling studies have been restricted to single layers of molecular data sets, i.e., comprising either gene expression or DNA methylation analyses [20, 32, 51].

In the present study, we employed similarity network fusion (SNF) analysis, which allows for the integration of multiple layers of large-scale molecular data sets [8]. We performed SNF analyses based on RNA sequencing transcriptomic and mass spectrometry (MS)-based proteomic profiling data of a large cohort of PAs to discern the biological heterogeneity of these tumors. As there is a known discordance between mRNA and protein expression [12, 23, 52], it was important to integrate the data to gain a better overview of potential intertumoral biological differences in PAs. Indeed, with pathways such as “Interferon Signaling” and “T Cell Receptor Signaling”, these data led us to the discovery that the profile of immune cells, which are part of the tumor microenvironment, may discriminate PAs into two biologically and clinically distinct groups, with Group 1 tumors being more frequently located in the supratentorial compartment, manifesting at younger age and being associated with less favorable progression-free survival.

## Materials and methods

### Patient samples

Tumor tissue samples from PA patients were obtained from the CNS tumor tissue bank Düsseldorf at the Institute of Neuropathology, University Hospital Düsseldorf, Germany, and from the Hospital de Santa Maria, Centro Hospitalar Universitário Lisboa Norte, in Lisbon, Portugal. Patients or parents provided their written informed consent for the use of the tissue samples for research purposes, in accordance with the requirements of the internal review boards. The study was approved by the Ethics Committee of the Medical Faculty, Heinrich Heine University Düsseldorf (study number: 5604). All samples analyzed in this study were collected from newly diagnosed patients and were flash-frozen directly after surgical resection. Each specimen used for protein and RNA extraction was histologically assessed to assure the presence of cellular tumor tissue with an estimated tumor cell content of > 70%. All tumors were histologically classified as PAs according to the criteria of the World Health Organization (WHO) classification of CNS tumors [21].

### Detection of *BRAF* gene alterations

Structural alterations in *BRAF*, i.e., *KIAA1549::BRAF* fusions were demonstrated in the diagnostic setting either by reverse transcription PCR or by in situ hybridization. Briefly, fusions were detected using the primers for the most common fusion products (*KIAA1549::BRAF* exons 15::9 or 16::9) and visualized using gel electrophoresis. Fusions were confirmed using Arriba v2.4.0 algorithm for samples with RNA sequencing [43]. Arriba was run with default settings against the hg38 reference genome with the GENECODE annotation. For *BRAF* V600 missense mutations, droplet digital PCR (ddPCR) was performed as previously published [47].

### RNA sequencing

Total RNA was isolated from the fresh frozen PA tissue samples using the Maxwell® RSC simply RNA Tissue Kit (AS1340, Promega, Walldorf, Germany). To prepare the barcoded libraries, 500 ng total RNA was processed using the TruSeq RNA Sample Preparation v2 kit (low-throughput protocol; Illumina, San Diego, CA, USA). Libraries were validated and quantified using either DNA 1000 or high-sensitivity chips on a Bioanalyzer (Agilent, Santa Clara, CA, USA). 7.5 pM denatured libraries were input into cBot (Illumina), followed by deep sequencing using the HiSeq 2500 (Illumina) for 101 cycles, with an additional seven cycles for index reading. Fastq files were imported into Partek Flow (Partek Incorporated, St. Louis, MO, USA). Quality analysis and quality control were performed on all reads to assess read quality and to determine the amount of trimming required (both ends: 13 bases 5´ and 1 base 3´). Trimmed reads were aligned against the hg38 genome using the STAR v2.4.1d aligner. Unaligned reads were further processed using Bowtie 2 v2.2.5 aligner. Finally, aligned reads were combined before quantifying the expression against the ENSEMBL (release 84) database using the Partek Expectation–Maximization algorithm. Partek Flow default settings were used in all analyses. RNA sequencing data have been deposited in the European Genome–Phenome Archive under the identifier EGAD00001009053 (https://web3.ega-archive.org/).

### Mass spectrometry

For MS-based proteome analyses, proteins were extracted from fresh frozen PA tissue. Tissues were homogenized in urea buffer with a TissueLyser (Qiagen, Hilden, Germany) and subsequent sonication. After centrifugation for 15 min at 14,000×*g* and 4 °C, supernatants were collected. Protein concentration was determined via Pierce 660 nm Protein Assay (Thermo Fischer Scientific) and 10 µg protein per sample were desalted through short electrophoretic migration at 50 V for 10 min on a 4–12% Bis–Tris polyacrylamide gel (#EC62352BOX, Novex NuPAGE, Thermo Fischer Scientific). After silver staining, the resulting protein band for each sample was cut out, destained, reduced, alkylated and digested with trypsin before peptide extraction via sonication. Peptides were dissolved and diluted with 0.1% TFA (v/v).

MS-based proteome analyses were performed as previously described [33]. In brief, 15 µL peptide solution per sample were analyzed on a nano-high-performance liquid-chromatography electrospray ionization mass spectrometer. The analytical system was composed of an RSLCnano U3000 HPLC coupled to a QExactive Plus mass spectrometer via a nano-electrospray ion source (Thermo Fischer Scientific). Injected peptides were concentrated and desalted at a flow rate of 6 µL/min using a trapping column (Acclaim PepMao C18, 2 cm × 100 µm × 3 µm particle size, 100 Å pore size, Thermo Fischer Scientific) with 0.1% TFA (v/v) for 10 min. Subsequently, peptides were separated at a constant flowrate of 300 nL/min over a 120 min gradient using an analytical column (Acclaim PepMap RSLC C18, 25 cm × 75 µm × 2 µm particle size, 100 Å pore size, Thermo Fischer Scientific) at 60 °C. Separation was achieved through a gradient from 4% to 40% solvent B [solvent A: 0.1% (v/v) formic acid in water, solvent B: 0.1% (v/v) formic acid, 84% (v/v) acetonitrile in water]. Afterwards, peptides were ionized at a voltage of 1,400 V and introduced into the mass spectrometer operating in positive mode. Mass spectrometry scans were recorded in profile mode at a range from 350 to 2000 *m*/*z* at a resolution of 70,000, while tandem mass spectra were recorded at a resolution of 17,500. Tandem mass spectra were recorded with a data-dependent Top10 method and 30% normalized collision energy. Dynamic exclusion was activated with a repeat count of 1 for 100 s.

Proteome Discoverer (version 1.4.1.14, Thermo Fisher Scientific) was applied for peptide/protein identification using Mascot (version 2.4, Matrix Science, London, UK) as a search engine employing the UniProt database (human; including isoforms; date 2016–03-01). A false discovery rate of 1% (*p* ≤ 0.01) at the peptide level was set as the identification threshold. Proteins were quantified with Progenesis QI for Proteomics (Version 2.0, Nonlinear Dynamics, Waters Corporation, Newcastle upon Tyne, UK). The mass spectrometry proteomics data have been deposited with the ProteomeXchange Consortium via the PRIDE partner repository (https://www.ebi.ac.uk/pride/) with the data set identifier PXD035773.

### DNA methylation profiling

Global DNA methylation data of 52 samples presented in this study were generated using tumor DNA extracted either from formalin-fixed paraffin-embedded tissue samples (FFPE, 32 tumors) or from flash-frozen tissue samples (FF, 20 tumors). Tumor DNA was hybridized to Illumina Infinium EPIC Methylation BeadChip Arrays. Methylation profiling was performed according to the manufacturer’s instructions at the DKFZ Genomics and Proteomics Core Facility (Heidelberg, Germany). All analyses were performed in Partek Genomic Suite (Partek Incorporated, St. Louis, MO, USA). FFPE and FF samples were processed individually and then combined following beta-value determination. The complete CpG methylation values have been deposited in NCBI’s GEO under accession number GSE210353. Normalization and generation of beta values were performed after NOOB background normalization. DNA methylation analysis using the CNS tumor methylation profiling classifier [7] confirmed the diagnosis of PA in 44 patients. In the remaining 8 patients, DNA methylation analysis revealed 4 control tissue samples, 3 samples with no matching methylation class and 1 sonic hedgehog medulloblastoma (histologically a PA with a BRAF-fusion).

### Similarity network fusion

This method has been described by Wang et al. [45]. Briefly, patient similarity matrices were constructed for each data type using Euclidean distance on samples that shared collected data for mRNA expression (48 samples, 13,498 features), proteome expression (43 samples, 2457 features) and methylation beta value (52 samples, 865,860 features) data sets. SNF was performed using 28 samples overlapping between mRNA and protein expression, 25 samples overlapping between mRNA and protein expression and methylation beta values, or individual data sets. SNF was run setting the number of nearest sample neighbors *K* = 10, the hyperparameter alpha = 0.5 and the number of iterations for the diffusion process *T* = 10. To obtain network clusters, spectral clustering was performed on networks representing each of the data types independently, as well as on the fused network to which the SNF process had converged. Analysis was visualized with Cytoscape (www.cytoscape.org) using the minimum number of entries that contained all samples based on the highest degree of relatedness.

### Hierarchical clustering and group extension

SNF group extension was conducted using genes with significant differential expression (*p* ≤ 0.05 and fold change ± 2) ranked by *p* value. The top 100, 50 and 25 up- and down-differentially regulated genes and proteins were used to generate signatures (Supplementary Tables 2 and 3). Gene and protein signatures were visualized using hierarchical clustering (HCL) after normalizing mean expression to 0 with a standard deviation of 1 and using Pearson’s dissimilarity algorithm and average linkage in Partek Genomics Suite. HCL was first performed with SNF-overlapping samples and then with all samples. Signatures with the lowest number of genes capable of maintaining accurate group associations (50 genes or proteins) were used for subsequent analyses.

### Submap analysis

Submap analysis was used to determine similarities between different data sets and was performed using the GenePattern analysis platform (https://cloud.genepattern.org/gp/pages/index.jsf). Data set files were ordered based on groups and class files provided group information. Defaults were used, except for the number of genes used to determine similarities (10,000 markers for transcriptome analysis or 2000 markers for proteome analysis and subclass association (SA) matrix was adjusted using the False Discovery Rate (FDR)).

### Calculating correlation and ratio between mRNA and protein expression

For each gene product, we calculated Pearson correlation coefficients between its normalized, centered, and log-transformed transcript and protein levels. The statistical significance of the correlation was assessed by *p* value. In addition, the divergence of protein and mRNA expression was measured by the protein/mRNA ratio. For all 2107 products, protein/mRNA ratios within each group were computed by dividing the median of the log protein and mRNA levels.

### Pathway analysis

Ingenuity pathway analysis (IPA, Qiagen) was conducted using genes with significant differential expression (*p* ≤ 0.05 and fold change ± 2). The significance cutoff for IPA was set to *p* ≤ 0.05 and an activation *z* score of ± 1.5. In addition, for upstream regulators, we filtered out biological drugs, all chemicals and miRNA entries.

Gene Set Enrichment Analysis (GSEA) was performed using the *t* values from the unpaired *t* tests for both mRNA and protein expression data. Gene sets were comprised of curated pathways from several databases, including GO, Reactome, KEGG (April_01_2019 version; http://download.baderlab.org/EM_Genesets/current_release/ Human/symbol/), and visualized using Cytoscape (www.cytoscape.org; main figure: *p* ≤ 0.0005, *q* ≤ 0.03, similarity cutoff 0.5; supplementary figure: *p* ≤ 0.001, *q* ≤ 0.05, similarity cutoff 0.5).

### Deconvolution analyses

ESTIMATE was carried out in R (version 4.0) using default parameters [50]. Briefly, data files were loaded and processed using the estimate package, identifiers were gene symbols and platforms were “illumina” for the RNA sequencing data sets and “affymetrix” for the microarray. Data were then visualized using GraphPad Prism (version 5.0) (https://www.graphpad.com/scientific-software/prism. Single-cell RNA sequencing data signatures were generated by Reitman et al*.* [34] and imported into CIBERSORT (https://cibersort.stanford.edu/) as a “signature matrix”. CIBERSORT was performed for each data set using default settings. Data were visualized using GraphPad Prism.

### Multiplex immunofluorescence

FFPE sections of PA patients were stained using Opal Polaris 7 colour kit (NEL861001KT, Akoya Biosciences, Inc.) based on thyramide signal amplification fluorescent immunohistochemistry. The staining targeting anti-human CD4 (1:50, MA5-16,338, Thermo Fisher Scientific), CD8 (1:150, M7103, DAKO), PD-1 (1:300, ab137132, Abcam), FoxP3 (1:200, DIA-FX3, Dianova), Iba-1 (1:450, 019-19741, WAKO) and vWF (1:120, A0082, DAKO) was performed on LabSat™ Research Automated Staining Instrument (Lunaphore Technologies SA). Whole slide multispectral scans were acquired at 0,5 µm/pixel on Vectra Polaris Imaging System using MOTiF™ technology (Akoya Biosciences, Inc.) and analyzed using HALO™ image analysis software (Indica Labs).

### Bioinformatic and statistical analyses

For validation purposes, ICGC data of 73 PAs [17] were downloaded from the European Genome–Phenome Archive (https://www.ebi.ac.uk/ega/datasets/EGAD00001000617) and processed in the same way as described for the discovery cohort. The processed and log_2_-transformed validation data from a further cohort of 191 PAs published by Kool et al. [5] were downloaded from the R2 Genomics Analysis and Visualization Platform (https://hgserver1.amc.nl/cgi-bin/r2/main.cgi). PA log ratio proteomic profiling data (*n* = 39) from PDC000180 was downloaded directly from the CPTAC data portal (https://cptac-data-portal.georgetown.edu/cptacPublic/). Statistical analyses were performed using Partek Genomic Suite or GraphPad Prism. *T* tests or Mann–Whitney tests (non-parametric *t* tests) were used for comparisons between two groups for statistical analysis. *χ*^2^ tests were performed to analyze clinicopathological traits. Differences between groups were considered statistically significant at* p* < 0.05. Kaplan Meier progression-free survival analyses were calculated using the log-rank method and multivariate analysis was calculated using the Cox regression method.

## Results

### Integrative multi-omic analysis of PA tissue samples

Our cohort consisted of a total of 62 flash-frozen primary PA tissue samples that were annotated with various clinical features (see clinical information in Supplementary Table 1). We employed an integrative multi-omics approach and performed DNA methylation, transcriptomic and proteomic measurements on 52, 48 and 43 partially overlapping samples, respectively (Supplementary Fig. 1). For proteomic analysis, we retained only proteins with at least three detected peptide ratios and with no missing values. Together, these stringent criteria led to the unambiguous quantification of 2456 proteins.

**Fig. 1:**
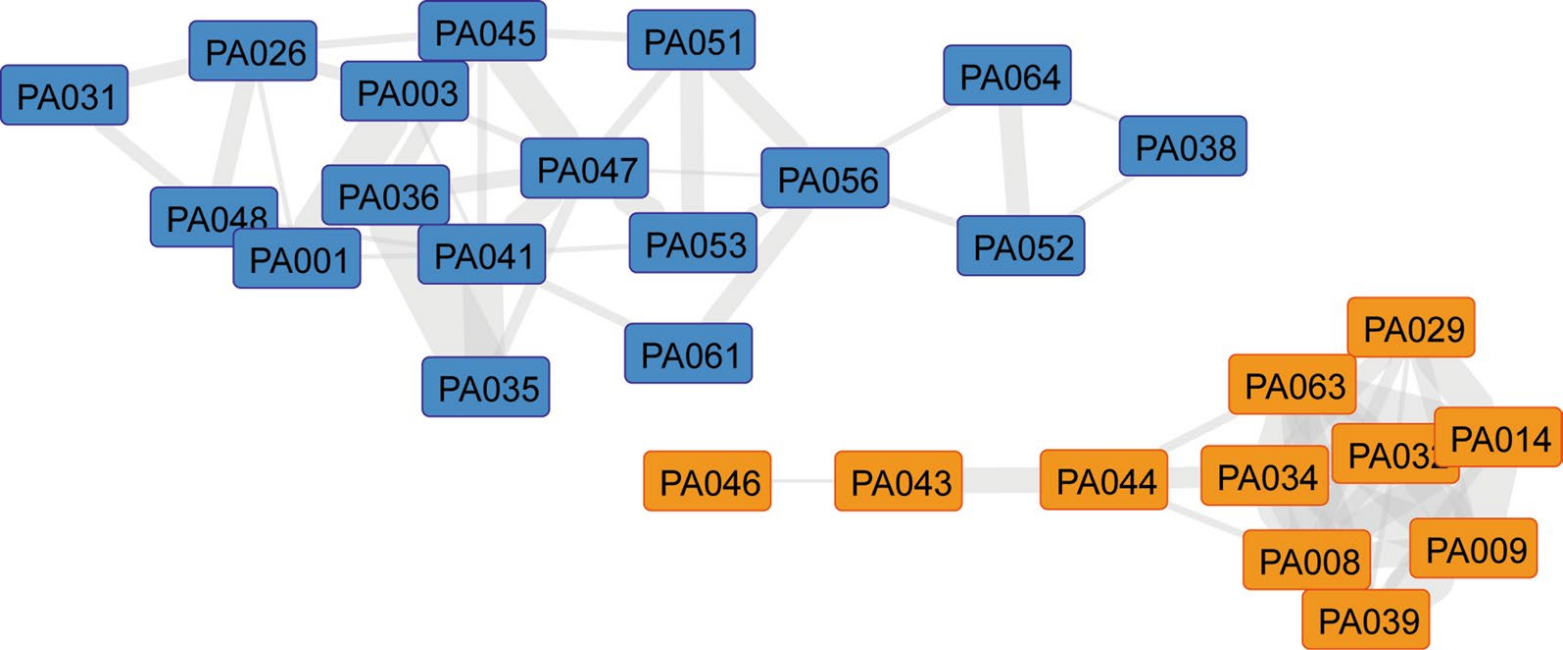
Similarity network fusion identifies two pilocytic astrocytoma groups using integrative multi-omics data. Similarity network fusion (SNF) representation clearly segregates two PA groups. Shorter edge length and greater thickness between samples (nodes) indicate more similarity

To uncover potential intertumoral heterogeneity in the investigated PA samples, we integrated transcriptomic and proteomic data sets (constituting 28 overlapping samples) using SNF [8, 12]. Strikingly, this efficiently identified two distinct tumor clusters that were designated as Group 1 and Group 2 (Fig. 1, Supplementary Fig. 2a). By contrast, separated SNF-based integrative clustering of each of the single omic layers, transcriptomics or proteomics, only poorly segregated groups (Supplementary Fig. 2b, c), highlighting the importance of combining multiple omics data. Moreover, integrating DNA methylation data to transcriptomic and proteomic data (for a total of 25 overlapping samples) did not further refine the SNF-based identification of patient groups (Supplementary Fig. 2d, e). On the contrary, this disrupted the initial group segregation obtained by transcriptomics and proteomics (Supplementary Fig. 2d, e, as compared to Fig. 1 and Supplementary Fig. 2a), arguing against the discriminatory power of DNA methylation to discern PA groups. Together, our integrative analyses of transcriptomics and proteomics revealed two PA groups.

### Validation of PA classification and clinical features of the PA groups

**Fig. 2:**
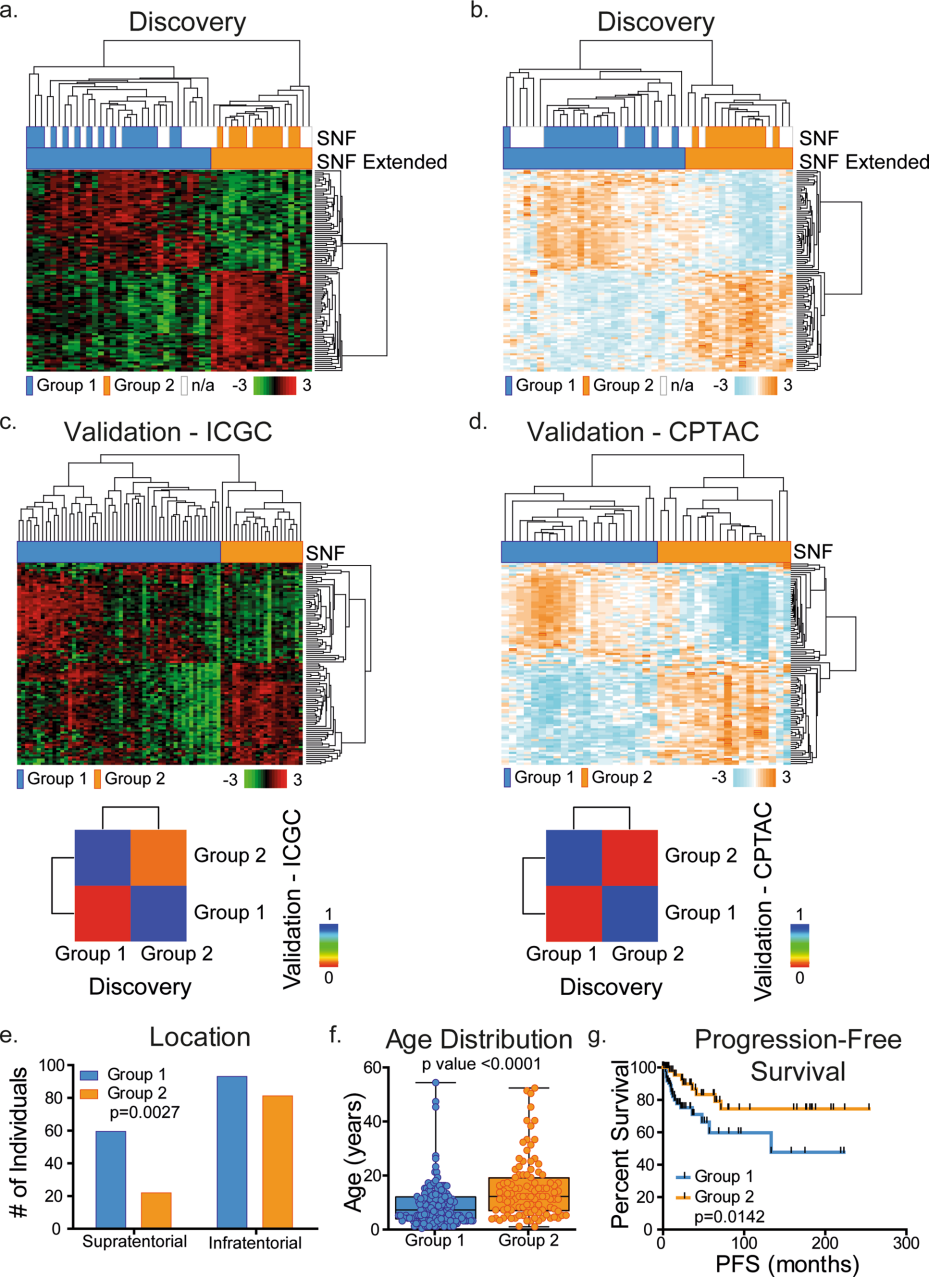
SNF groups are recapitulated in an extended PA and PA validation cohorts. **a**, **b** Hierarchical clustering of 100 gene/protein signatures based on *p* value allows for the expansion of groups to non-overlapping RNA sequencing (**a**) and mass spectrometry (**b**) samples. **c**, **d** 100-gene/protein signature applied to non-overlapping transcriptomic (ICGC) [17], **c** and proteomic (CPTAC) [31], **d** validation cohorts segregate samples into two groups. Lower panel, submap analyses show close relatedness between discovery and both validation cohorts. **e**–**g** Combined data set analysis of clinical features shows that the majority of younger patients belong to Group 1 and adults belong to Group 2. (*p* < 0.0001, Mann Whitney test, **e**), location shows an enrichment of infratentorial regions for Group 2 (*p* = 0.0024, Fisher’s Exact test, **f**), and Kaplan–Meier plot shows patients in Group 1 are more likely to develop recurrent tumors (*p* = 0.0142, log rank method, **g**)

To further extend our findings to the non-overlapping samples of our cohort and validate these in independent cohorts, we generated a gene and a protein signature capable of distinguishing between the two identified PA groups, as calculated by SNF clustering. These signatures were determined by extracting the most differentially expressed genes or proteins between Group 1 and Group 2, Resulting in 100 validated gene and 100 validated protein signatures (see Supplementary Tables 2 and 3). These allowed us to recapitulate our PA classification using only single omics data. We first assessed the validity of the PA classification using 20 additional PA samples that were subjected to RNA sequencing and 15 additional PA samples profiled by proteomic analysis from the same original cohort, but that had not been used in the integrative multi-omics analysis. Semi-unsupervised clustering analysis of transcriptomic data of this extended PA cohort (48 samples total) segregated the same groups as originally identified (Fig. 2a), further reinforcing these PA groups. Applying the same type of analysis to proteomic data of the extended cohort similarly led to the identification of the same PA groups (Fig. 2b), showing that using either a gene or a protein signature alone was sufficient to stratify PAs in an extended patient cohort. We next determined whether the patient stratification could be validated in non-overlapping PA cohorts. To this aim, we used RNA expression data from the International Cancer Genome Consortium (ICGC) (*n* = 73) [17] and Kool et al*.* (*n* = 191) [5] cohorts and protein expression from the Clinical Proteomic Tumor Analysis Consortium (CPTAC) (*n* = 65) [31]. A semi-unsupervised clustering analysis of these data using the same 100-gene or protein signature was conducted which led to the segregation of two groups in all cohorts (Fig. 2c, d, Supplementary Fig. 3). These were highly similar to the ones originally defined in our discovery cohort as Group 1 and Group 2, as measured by submap analysis (Fig. 2c, d, Supplementary Fig. 3).

After combining all four PA cohorts for a total of 365 PA samples, differences in relevant clinical parameters were analyzed between Group 1 and Group 2. This did not reveal any differences in gender distribution, *BRAF* mutation rate or *KIAA1549::BRAF* fusion occurrence between the two groups (Supplementary Fig. 4a, b). However, tumor location was different between groups, with more tumors in Group 1 being located in the supratentorial compartment, as compared to the preference of infratentorial tumors in Group 2 (*p* value = 0.0027; Fig. 2e). A more detailed analysis revealed that Group 1 tumors were either exclusively observed in optic pathway system and that Group 2 tumors were preferentially observed in the posterior fossa (*p* value = 0.0011; Supplementary Fig. 4c). In addition, the age of patients was significantly different between the two groups, with Group 1 patients being younger than Group 2 patients (mean ages were 7 years versus 12 years, respectively; *p* value < 0.0001; Fig. 2f), potentially corresponding to a younger and an older PA group. Remarkably, Group 1 patients exhibited reduced progression-free survival compared to Group 2 patients, highlighting the clinical significance of the identified groups (*p* value = 0.0142; Fig. 2g). This association was independent of age or tumor location, as determined by multivariate analysis (Supplementary Fig. 4d). Indeed, when we analyzed progression-free survival differences of the two subgroups, we observed no significant survival differences depending on the detailed tumor location. (Supplementary Fig. 5). Altogether, our analyses in additional cohorts confirmed the identification of two PA groups, and highlight that these likely discriminate younger versus older PA patients with distinct progression-free survival.

### Posttranscriptional regulation and pathway analysis of PA groups

**Fig. 3:**
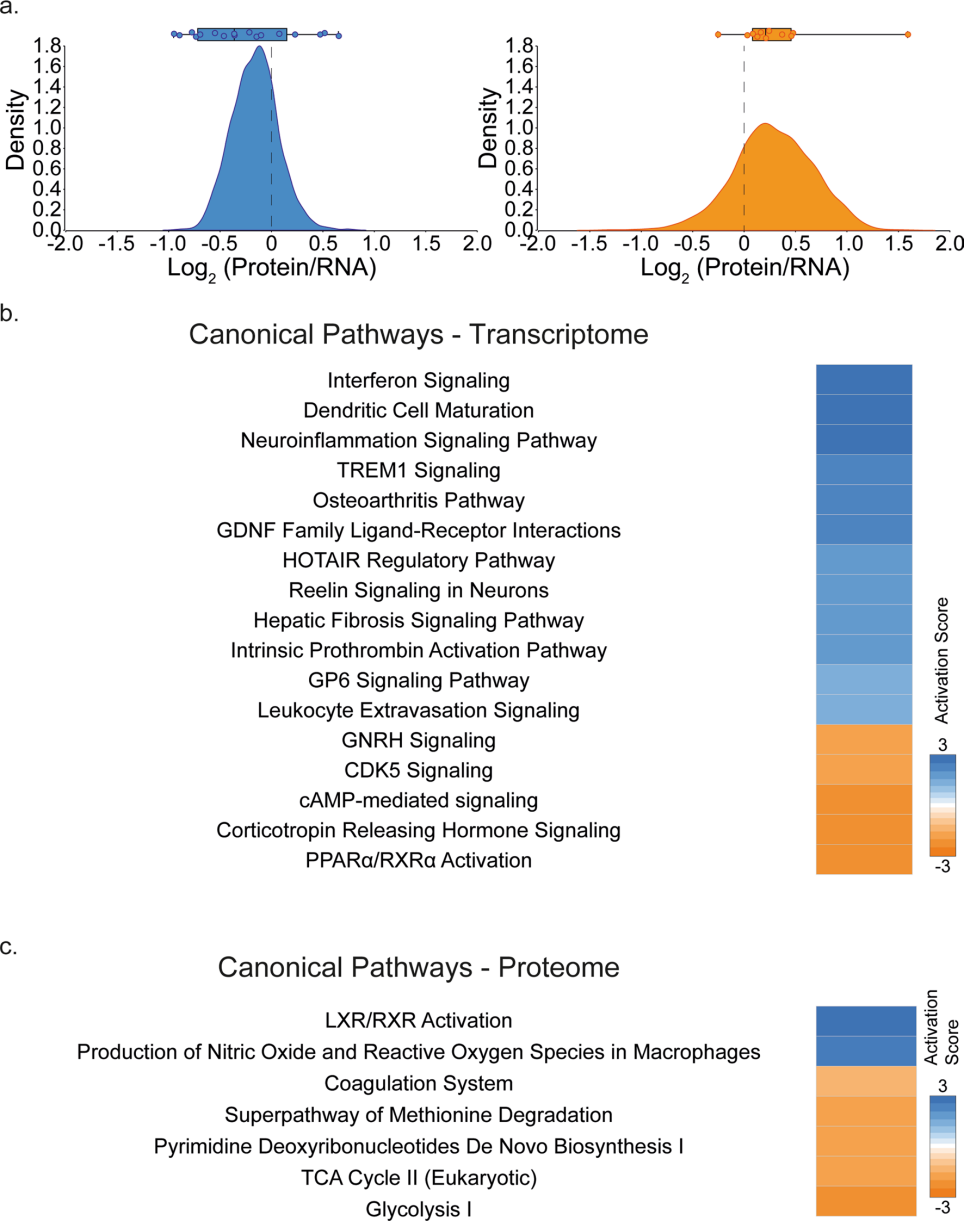
Ingenuity pathway analysis identifies differential canonical pathway activation between PA groups.** a** Distribution of protein/mRNA ratios in PA groups. Both groups display a non-centric ratio distribution (Mann Whitney test; *p* value = 0.0144), with an imbalance in favor of mRNA for Group 1 and protein for Group 2. Dotplots show the median protein/mRNA ratios for individual samples. **b**, **c** Group 1 PA were compared to Group 2 PA for both transcriptome (**b**) and proteome (**c**). Significant genes (fold change ± 2 and False Discovery rate *q* value < 0.05) were processed using IPA and significantly activated canonical pathways are shown. (Activation *z*-score ± 2, – log_10_ (*p* value) > 1.30.)

Given that transcript and protein expression levels are poorly correlated in primary tumors [12, 23, 52], we assessed their level of correlation in PAs and checked whether this level is group-specific. To this end, we computed pairwise Pearson test correlations for 2102 matched mRNA–protein pairs extracted from the original 28 samples of our discovery cohort. In line with previous studies, we uncovered a median Pearson correlation coefficient of 0.168, suggesting the occurrence of posttranscriptional mechanisms regulating gene expression in PA. To further explore such discrepancies between transcript and protein levels in this disease, we calculated the ratio of relative expression of protein and mRNA for each individual pair in each group. We observed that Group 1 and Group 2 displayed remarkably distinct distributions of such a ratio. While the protein/mRNA ratio is mainly distributed towards transcript expression in Group 1, it is the opposite in Group 2, for which protein expression is prevalent (Fig. 3a). This highlights that posttranscriptional regulation is group-related, suggesting different modes of control of gene expression in Group 1 versus Group 2 PA.

**Fig. 4:**
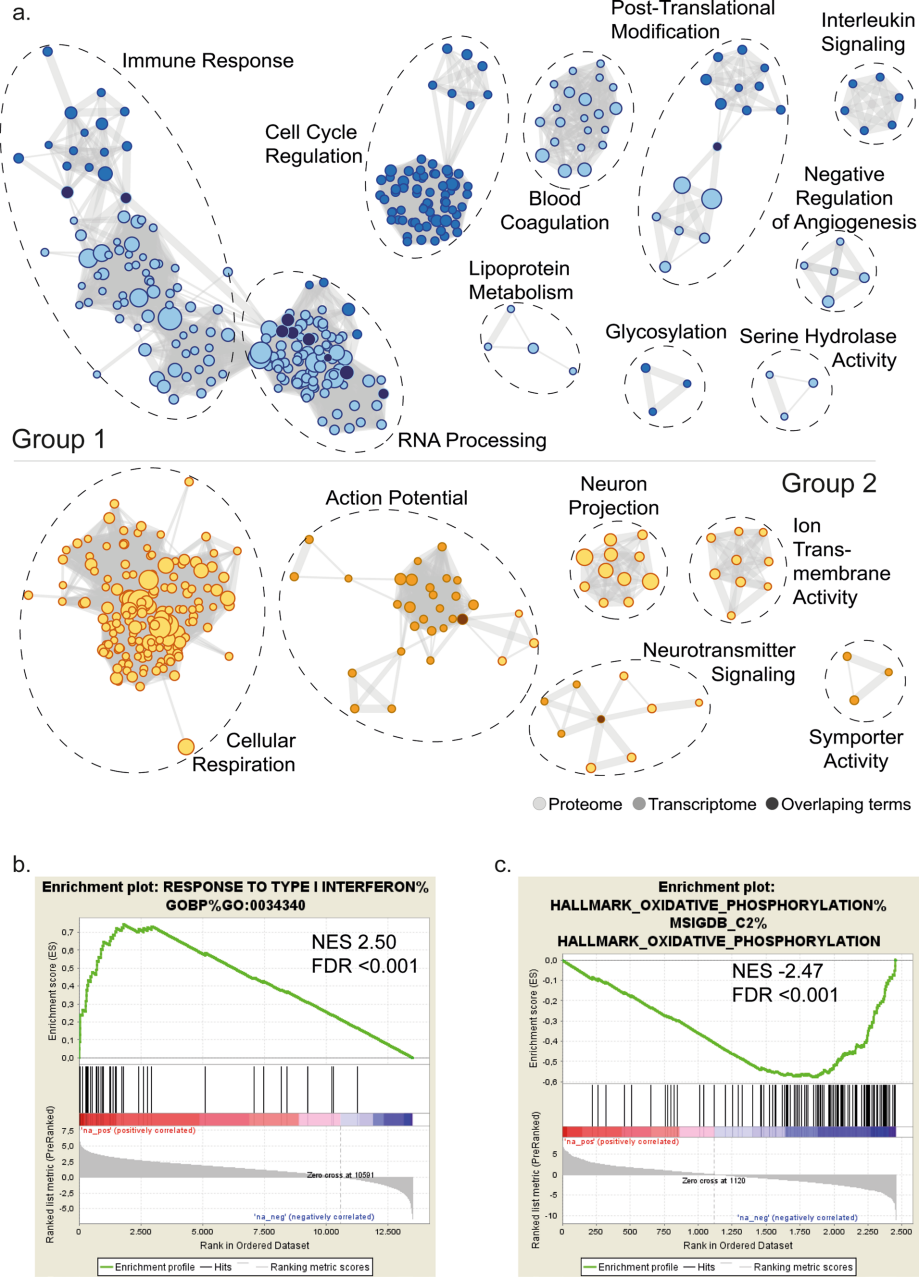
Integrative multi-omics identifies highly divergent pathways.** a** GSEA-based enrichment map representations based on groups (*p* value = 0; FDR < 0.03). Nodes (circles) represent enriched pathways identified on the basis of proteome or transcriptome expression, which are light or medium colored, respectively, and dark nodes represent overlapping terms between transcriptome and proteome expression. Edges connect pathways/nodes that share at least half of the terms defining them. Nodes grouped according to functional families are indicated on each network. **b**, **c** Enrichment plots of top transcriptomic gene set for Group 1 showing Response to Type I Interferon (**b**) and top proteomic gene set for Group 2 showing Hallmark Oxidative Phosphorylation (**c**)

To address the biological heterogeneity of PAs, we analyzed biological pathways that are active in each of the two groups. This was achieved by performing two types of complementary analyses: Ingenuity pathways analysis (IPA) and gene set enrichment analysis (GSEA). Using our transcriptomic data, we uncovered that Group 1 and Group 2 are characterized by distinct biological pathways. In particular, IPA showed that “interferon signaling”, “dendritic cell maturation” and “neuroinflammation signaling pathway” are specifically active in Group 1, while “gonadotropin-releasing hormone (GNRH)” and “cyclin-dependent kinase 5 (CDK5) signaling” are active in Group 2 (Fig. 3b and Supplementary Table 4). In keeping with the IPA, GSEA revealed an overrepresentation of immune response pathways (Fig. 4a and Supplementary Tables 6–7)—in particular of “Interferon Signaling” (top term, Fig. 4b and Supplementary Table 6) and “T Cell Receptor Signaling”—in Group 1. This was confirmed in the ICGC [17] and Kool et al. [5] cohorts (Supplementary Fig. 6), further reinforcing the importance of such pathways in this group. In addition, Group 1 was enriched for cell cycle and RNA processing-related pathways (Fig. 4a), while Group 2 was characterized by enrichment for action potential and neurotransmitter signaling pathways, as determined by GSEA (Fig. 4a). Supporting the discovery analysis, enrichment for action potential was confirmed in Group 2 using the ICGC and Kool et al. cohorts (Supplementary Fig. 6).

Notably, Group 1 was enriched for immune response pathways and, in particular, for “Interferon Signaling”, as well as “RNA Processing Pathways” and “Post-Translational Modification” (Fig. 4a) using GSEA of our proteomic data. However, “TCA Cycle II (Eukaryotic)” and “Glycolysis I”, among others, were pathways observed in the IPA using the proteomic data of Group 2 samples (Fig. 3c and Supplementary Table 5). These observations were verified using the discovery and validation—CPTAC GSEA. Specifically, cellular respiration (Fig. 4a and Supplementary Table 7) and “Oxidative Phosphorylation”, the top gene set (Fig. 4c) in the proteomic analysis, were verified using the validation proteomic data set (Supplementary Fig. 7). Therefore, integration of multiple data layers is required to observe the biological heterogeneity of PA.

### Distinct immune cell signatures in PA groups

To further investigate our observations of increased immune response gene signature in Group 1, we evaluated the level of immune cell infiltration in Group 1 versus Group 2 samples using the algorithm “Estimation of STromal and Immune cells in MAlignant Tumours using Expression data” (ESTIMATE) [50]. ESTIMATE scores are used to determine tumor purity; however, the ESTIMATE algorithm can also assess the presence of stromal and infiltrating immune cells. Both RNA sequencing data sets (Discovery and Validation—ICGC) showed a significantly higher immune score for Group 1 compared to Group 2, which was not evident in the microarray validation set (Validation-Kool et al. [5]) (Supplementary Fig. 8), possibly reflecting the limited resolution of microarray-based expression profiling compared to RNA-sequencing. Since Reitman et al*.* [34] showed that microglia, macrophages and T cells are present along with the PA tumor cells using single cell RNA sequencing, we used this information to create a signature file for each of these four cell types. By processing and analyzing our discovery, Validation-ICGC [17] and Validation-Kool et al. [5] data sets using CIBERSORT [26], we uncovered that, in the RNA sequencing data sets (Discovery and Validation—ICGC), T cells were specifically enriched in Group 1 compared to Group 2, while there was no difference in microglia or macrophages (Supplementary Fig. 9).

To confirm the previous results, we performed multiplex immunofluorescence on a subset of cases (*n* = 24). CD4, CD8 or IBA-1 positive cells were similarly represented between PA Groups 1 and 2 (Supplementary Fig. 10a–c). When we analyzed the immunosuppressive T cell populations, although FOXP3^+^CD4^+^ cells were not different between the two groups, exhausted T cells (PD1^+^CD8^+^) cells showed a strong trend towards being able to discriminate Group 1 from Group 2, Group 2 having a higher percentage of PD1 positive CD8^+^ T cells (Supplementary Fig. 10d–f).

## Discussion

Genome-wide profiling and next-generation-based sequencing approaches have provided profound insights into the pathomechanisms underlying PA development. In particular, these methods have revealed that aberrant activation of the MAPK pathway constitutes a hallmark feature of these tumors [6]. Oncogenic activation of MAPK is caused by alterations affecting *BRAF*, *NF1*, *FGFR1* or, rarely, other MAPK pathway genes [17, 24, 32]. However, further biological stratification of this disease has not evolved in the past decade, in contrast to other brain tumor entities in children and adults [8, 30, 41, 44]. Only a limited number of studies have suggested intertumoral heterogeneity of the disease as identified by distinct transcriptomes [20, 39, 48], DNA methylomes [20, 34, 36, 51], ploidy [11] or DNA copy number alterations [14, 32]. Importantly, none of these studies reported reproducible features of the identified subgroups. Furthermore, high-resolution DNA methylation profiles used for the molecular neuropathology classifier [7] are very accurate in dissecting molecular entities, with the important exception of LGG, including PA. At present, the algorithm identifies PA predominantly based on anatomic location. Therefore, currently available approaches fail to differentiate clinically relevant groups of the disease, suggesting either that such PA groups do not exist or that single layer omics approaches insufficiently discriminate the biological heterogeneity of the disease.

Thus, we decided to apply an innovative, integrative multi-omics approach, which has already provided fundamental insights into the tumor biology of breast cancer [2, 19], pancreatic ductal adenocarcinoma [6], glioblastoma [46], and medulloblastoma [3, 12], among other entities. Our bioinformatics approach integrates proteomic, transcriptomic, epigenomic and mutational profiles. Subsequently, SNF provided compelling evidence for the existence of two core PA groups using combined proteomic and transcriptomic data in an institutional discovery cohort. Since integrative multi-omics classification requires generating multiple data sets, which is difficult in the clinical setting due to sample quality and/or financial constraints, we established highly accurate classification approaches using only RNA- or protein-based signatures. Notably, by applying this strategy to three independent non-overlapping validation cohorts, we confirmed the existence of the two core PA groups.

Our integrative multi-omics data show highly distinct pathway enrichment according to PA group. Most importantly, immune response and associated pathways, including “Interferon signaling”, “Antigen Processing and Presentation”, “Cellular Response to Tumor Necrosis Factor”, and “T Cell Receptor Signaling Pathway” (Fig. 3, Supplementary Table 6), were significantly overrepresented in Group 1. Notably, this association could be confirmed using both transcriptomic validation cohorts. Furthermore, we uncovered an enrichment for the T cell gene signature in Group 1, likely pointing to a higher infiltration of T cells in Group 1 versus Group 2 PA. This is in agreement with previous reports that detected T cells within PA tumor tissues using histology [35] or single-cell RNA sequencing [34] while being unable to highlight intertumoral heterogeneity for this parameter. PD1 is a marker of T cell exhaustion [25] and our data suggest an exhausted phenotype of CD8^+^ T cells in Group 2 which is consistent with the lack of observed immune activation at the transcriptomic or proteomic levels in this group. Consistent with our bioinformatics analyses, we did not observe differences in other immunosuppressive populations including regulatory T cells and macrophages.

In our proteomic analysis, we were also able to confirm that Group 2 had greater enrichment of gene sets involved in Cellular Respiration, such as “Oxydative Phosphorylation”, “Mitochondrial Respiratory Chain” and “The Citric Acid (TCA) Cycle and Respiratory Electron Transport” using our discovery and validation cohorts. These data are in line with high rate of mitochondrial mutations detected by Leuth et al., where they observed that 53% of the mutations in PA tumors had mutations in genes involved in oxidative phosphorylation pathways [22]. The dysregulated oxidative phosphorylation pathway may enhance reactive oxygen species accumulation and lead to either an increase in proliferation rate or a decrease in apoptotic activity thereby potentially enhancing tumor growth.

Furthermore, “RNA processing” and associated pathways were remarkably divergent between the two PA groups. Notably, we demonstrated that, in general, pathway regulation was predominantly driven by RNA signatures in Group 1, while proteomic-based pathway regulation was significantly increased in Group 2, based on our integrative multi-omic discovery cohort. In addition, “RNA processing” and associated pathways were among the most consistently affected pathways in both transcriptomic validation cohorts.

Finally, we were able to delineate distinct clinical features according to the PA groups using the combined discovery and validation data sets. Group 1 tumors were evenly distributed between the supra- and infratentorial compartments, while Group 2 PAs were more commonly located in the infratentorial region. In addition, age distribution differs significantly, as patients with Group 1 PAs were significantly younger than those with tumors in Group 2. Finally, it has previously been reported that infratentorial tumors are associated with better progression-free survival [38]. While we found a difference in progression-free survival between Group 1 and Group 2, this was independent of tumor location. Importantly, we observed improved progression-free survival in Group 2, which was unexpected, because adults with PA have been reported to have worse prognoses than children [15, 42, 49]. Taken together, our data thus provide novel insights into the biological heterogeneity of PA, which may allow for more accurate biological disease stratification.

## Supplementary Information

Below is the link to the electronic supplementary material.

Supplementary file1 (PDF 2769 KB)

Supplementary file2 (XLSX 824 KB)

## Data Availability

The data that support the findings in this study have been deposited in separate repositories. RNA sequencing data has been deposited in the European Genome-phenome Archive under the identifier EGAD00001009053 (https://web3.ega-archive.org/). The mass spectrometry proteomics data has been deposited with the ProteomeXchange Consortium via the PRIDE partner repository (https://www.ebi.ac.uk/pride/) with the data set identifier PXD035773. The complete CpG methylation values have been deposited in NCBI’s GEO under accession number GSE210353.
