# Supplementary material for: Integrative multi-omics reveals two biologically distinct groups of pilocytic astrocytoma
Source: Acta Neuropathol. 2023 Sep 1;146(4):551–64. doi: 10.1007/s00401-023-02626-5 (PMC10500011; doi:10.1007/s00401-023-02626-5)
Supplement: Supplementary file 1 — Supplementary file1 (PDF 2769 KB) [file 401_2023_2626_MOESM1_ESM.pdf]

Picard Supplementary Figure 1

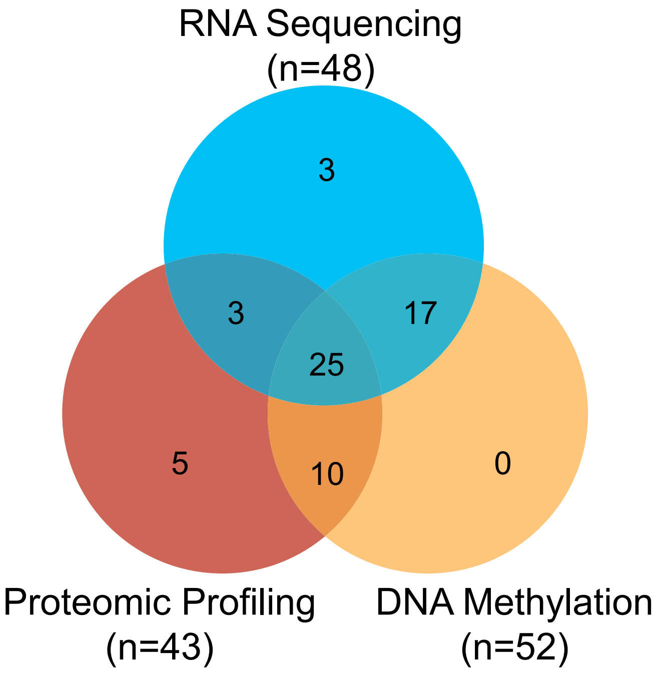

Picard Supplementary Figure 2

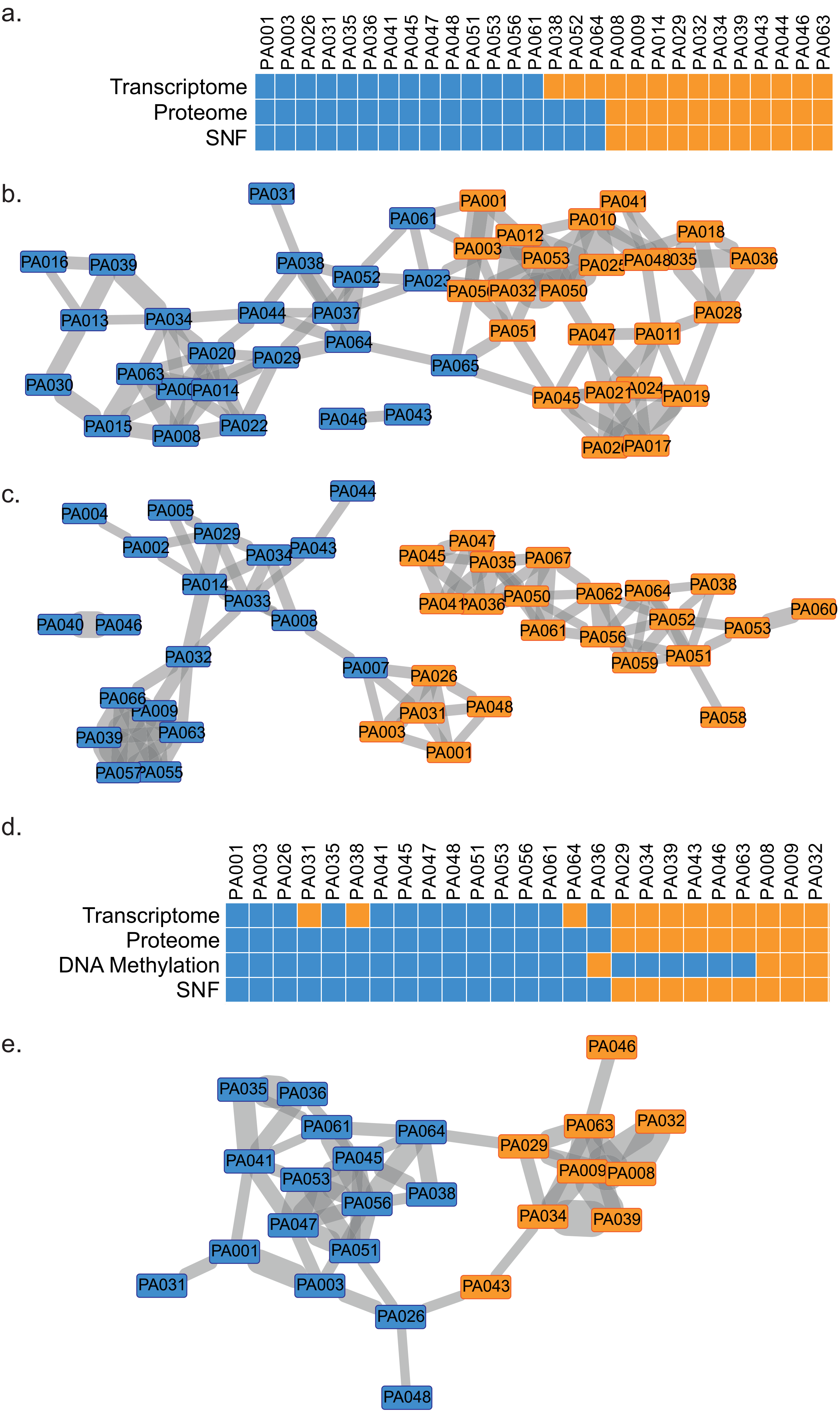

Picard Supplementary Figure 3

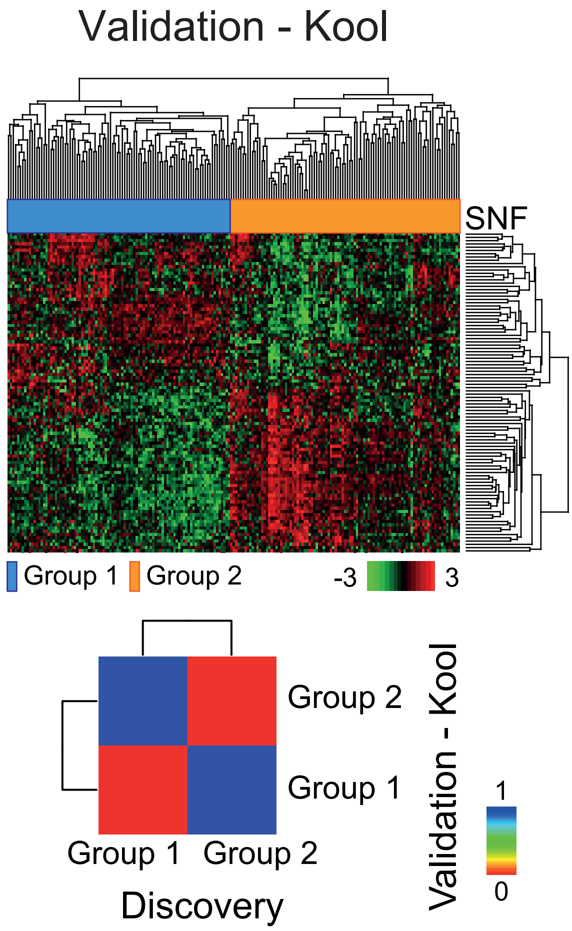

Picard Supplementary Figure 4

a.

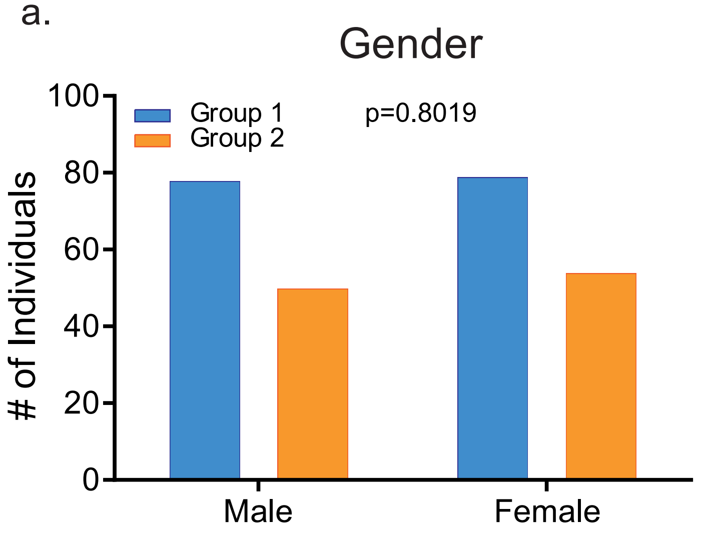

b.

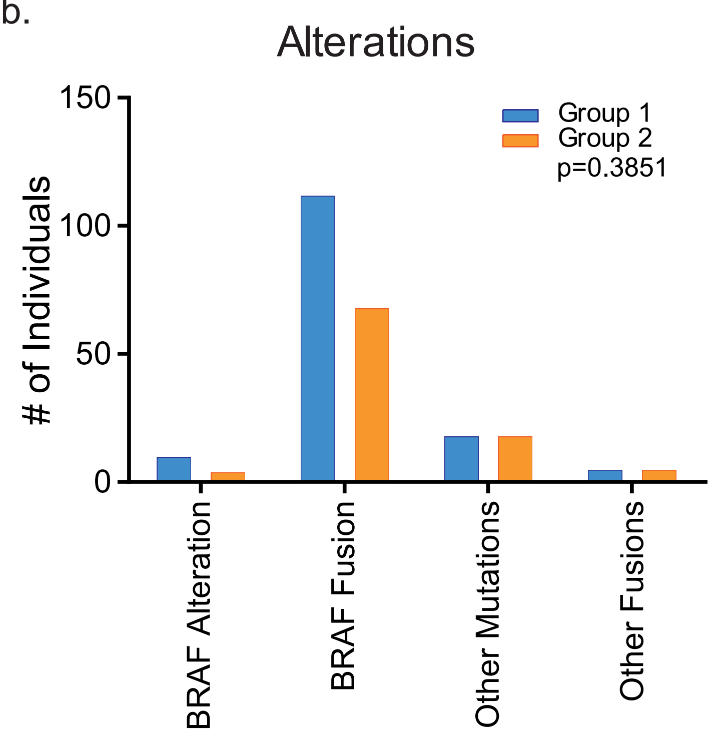

c.

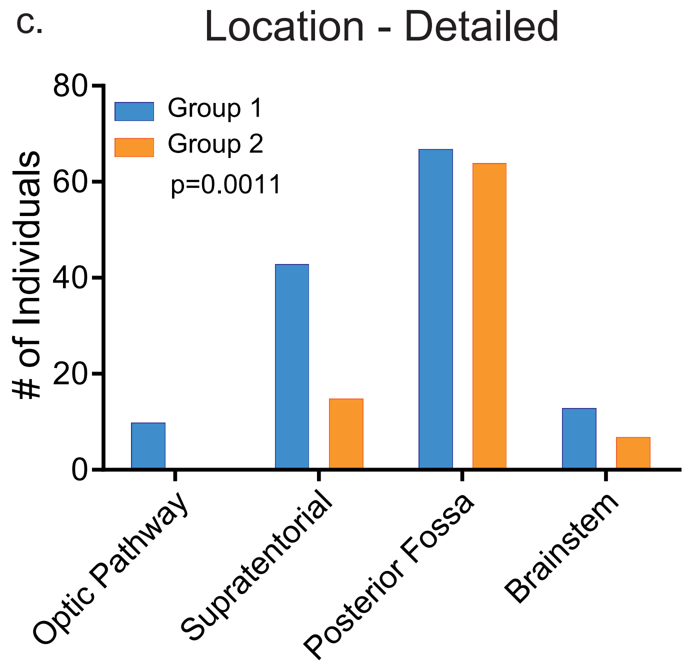

d.

Factor	Hazard Ratio	95% CI	Significance
PA Group	0.365	0.158-0.844	0.018
Age Group	2184.359	$0\text{-}1.919\times10^{64}$	0.914
Alteration Type	0.244	0.037-1.635	0.146
Gender	0.923	0.145-5.889	0.933
Location	2779.621	$0\text{-}3.551\times10^{36}$	0.838

Picard Supplementary Figure 5

a.

Factor	Hazard Ratio	95% CI	Significance
Location - Detailed	0.949	0.282-3.201	0.933

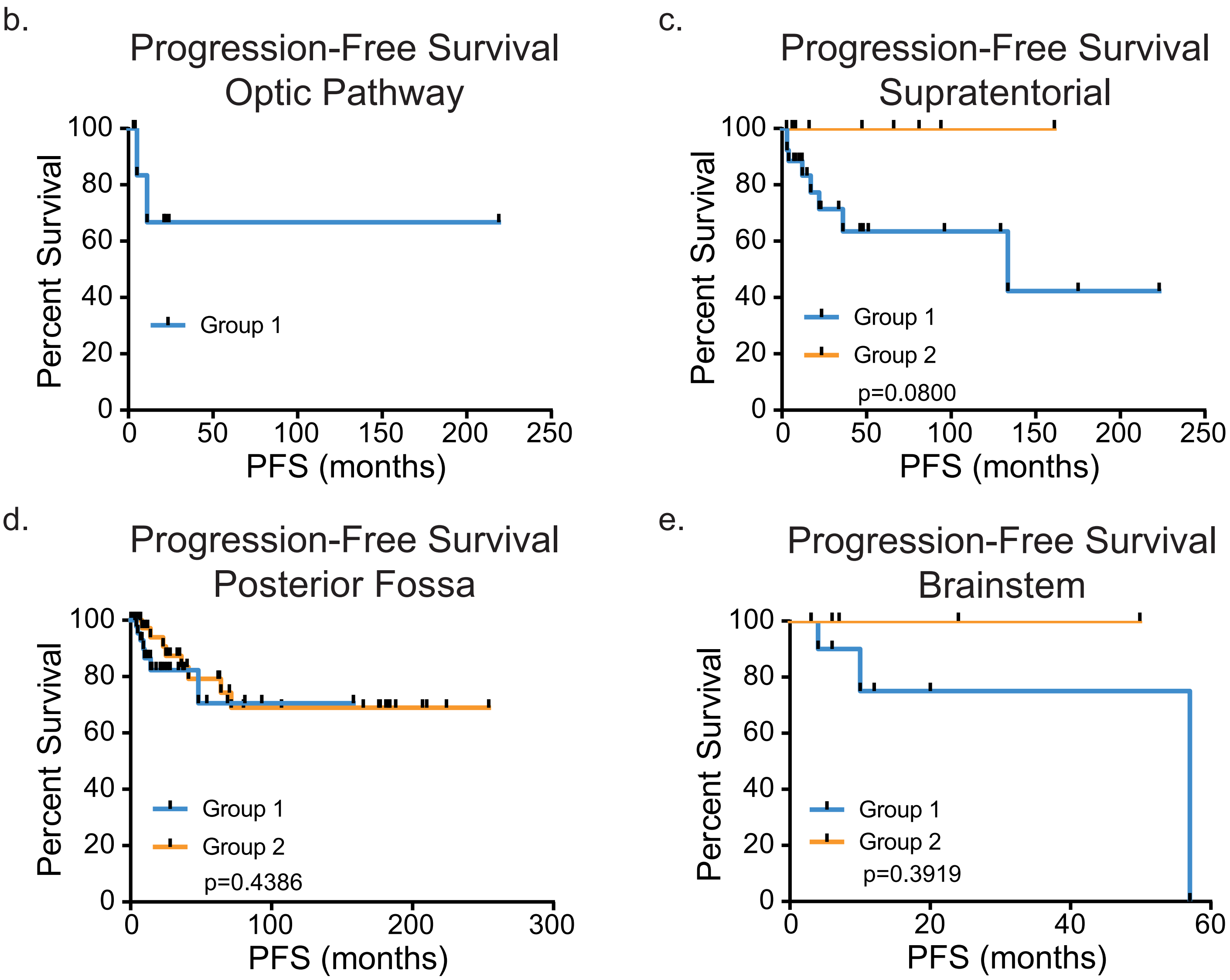

Picard Supplementary Figure 6

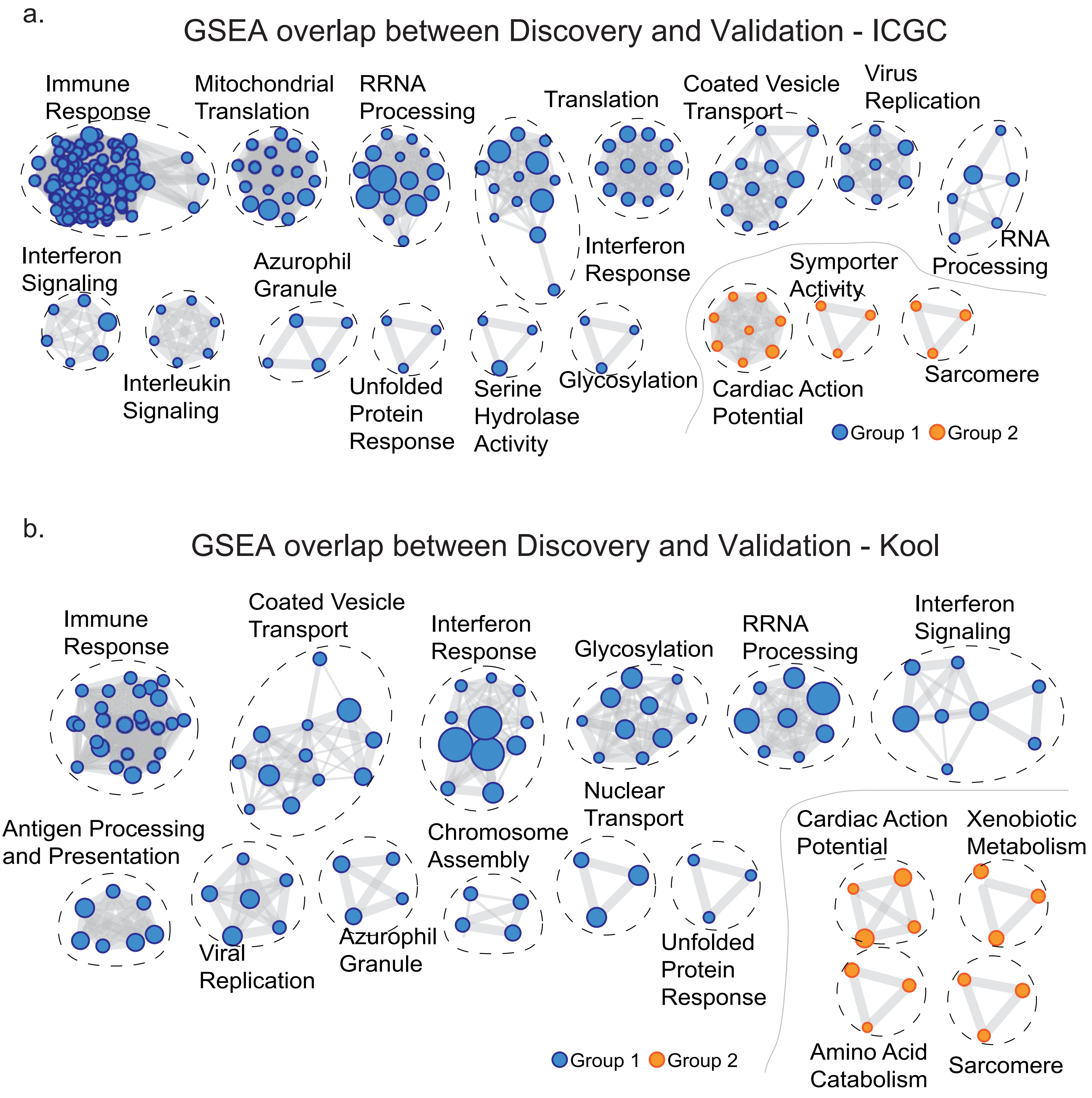

Picard Supplementary Figure 7

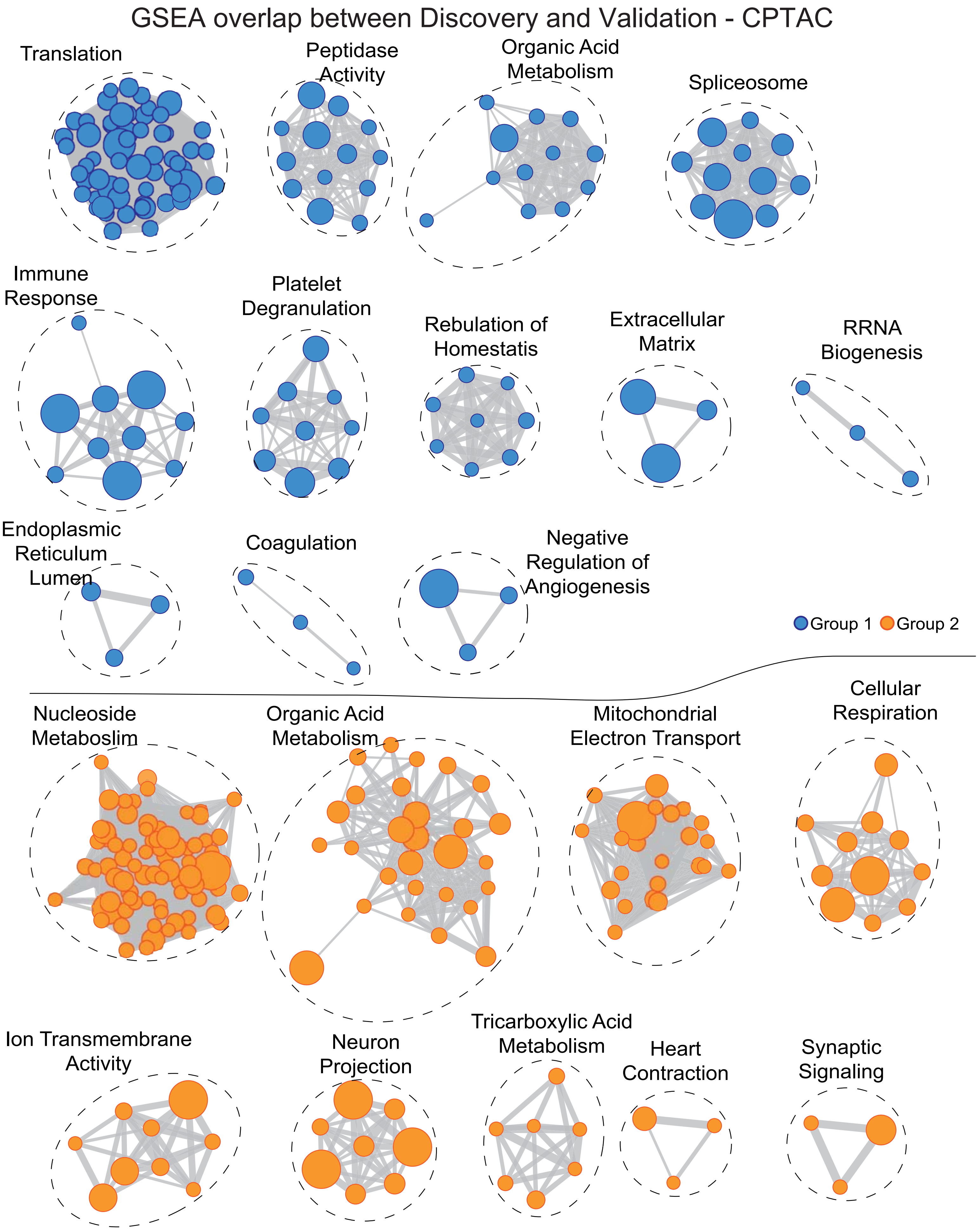

Picard Supplementary Figure 8

a.

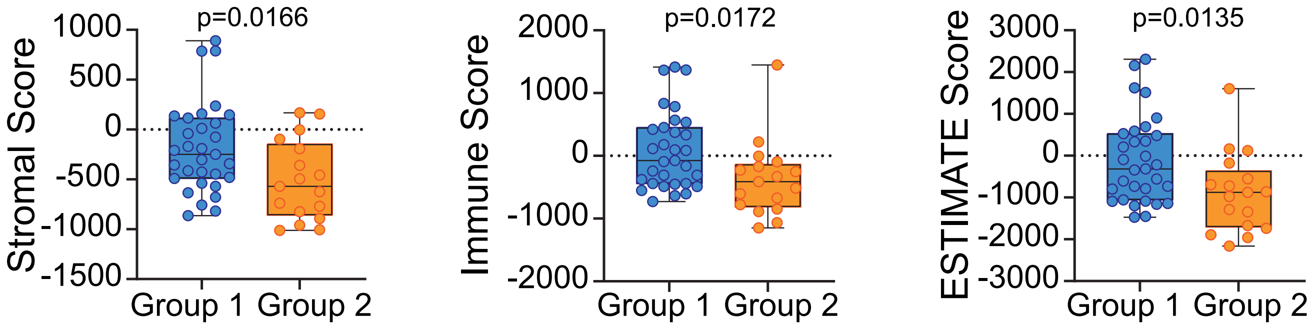

b.

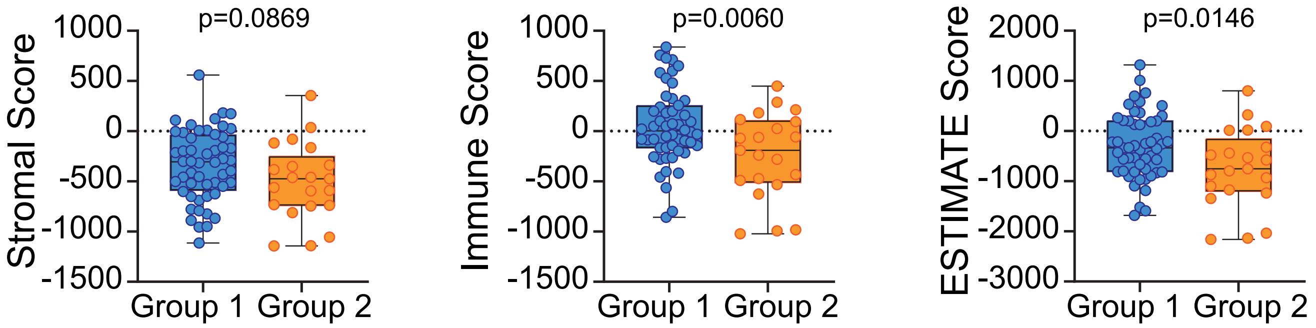

c.

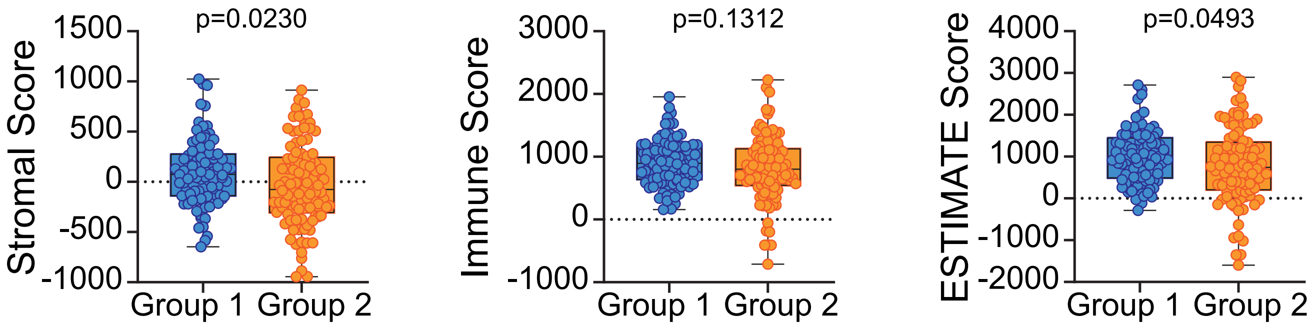

Picard Supplementary Figure 9

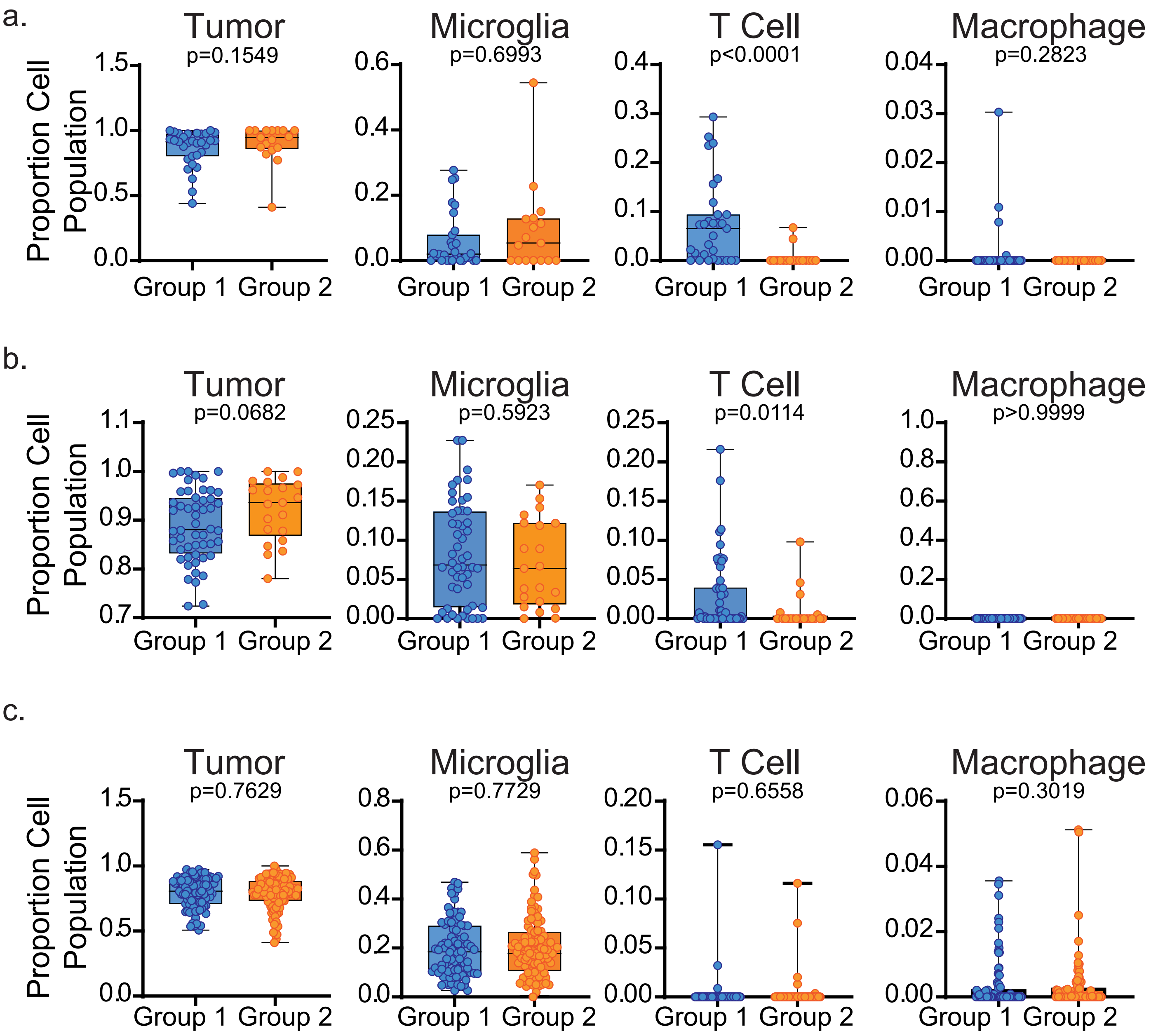

Picard Supplementary Figure 10

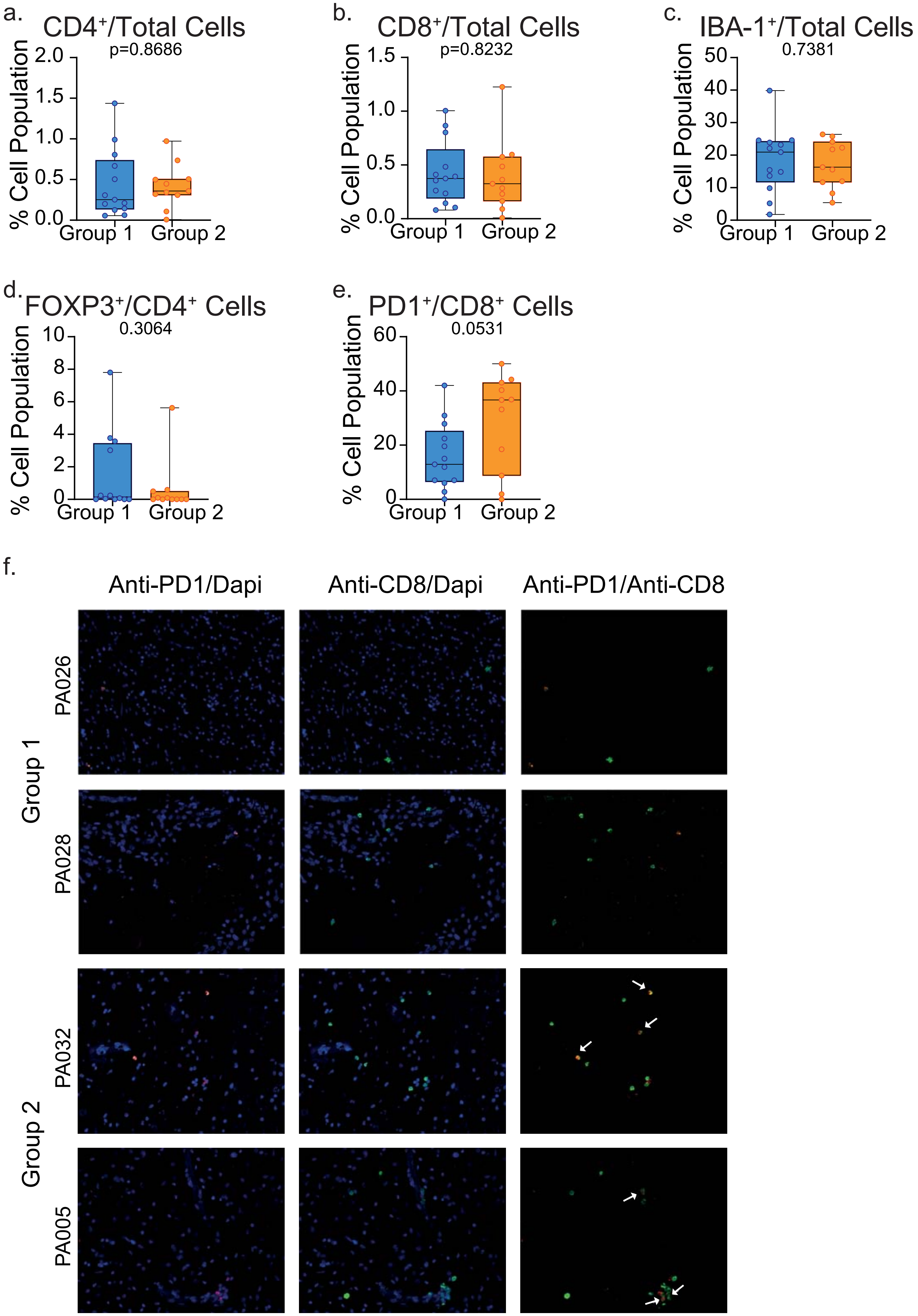

Supplementary Figure Legends

Supplementary Figure S1. Distribution of PAs processed using RNA sequencing, mass spectrometry and methylation profiling. Venn diagram displaying the overlap between the different omics platforms.

Supplementary Figure S2. Similarity network and network fusion analyses of PA show two groups using multilayer proteogenomic data. a. Similarity network clustering of individual data layers of PA samples and similarity network fusion (SNF) clustering integrating all data layers are shown in the bottom row. **b-c.** Similarity network representations of transcriptome (**b.**) and proteome (**c.**) clustering show incomplete separation of PA groups. Shorter edge length and greater thickness between samples (nodes) indicates more similarity. **d.** Similarity network clustering of individual data layers of PA samples, including transcriptome, proteome and DNA methylation, and SNF clustering integrating all data layers is shown in the bottom row. **e.** SNF representation incompletely segregates samples into both PA groups. Shorter edge length and greater thickness between samples (nodes) indicates more similarity.

Supplementary Figure S3. SNF groups are recapitulated in a micro array validation cohort. 100-gene signature applied to non-overlapping transcriptomic (Kool et al.) validation cohort segregates samples into two groups. Lower panel, submap analyses show close relatedness between discovery and validation cohort.

Supplementary Figure S4. Clinical analyses of PA groups in discovery and validation cohorts. a-b. Mann-Whitney test was performed on gender (**a.**, p=0.8019) and genetic alterations (**b.**, p=0.3851) for the combined grouped samples. **c.** Combined dataset analysis of detailed location which separates optic pathway from supratentorial and infratentorial is subdivided into posterior fossa and brainstem. Location shows an

enrichment of Group 1 samples in both the optic pathway and supratentorial regions (p=0.0011, Mann-Whitney test). **d.** Multivariate analysis of PA clinical and pathological characteristics.

Supplementary Figure S5. Detailed location analyses of PA groups in discovery and validation cohorts. a. Multivariate analysis of detailed location and PA subgroups shows no benefit for survival. **b-e**. Kaplan-Meier progression-free survival curves show no significant survival difference between PA Group 1 and Group 2.

Supplementary Figure S6. Gene Set Enrichment Analysis overlap of discovery and validation RNA Sequencing cohorts. a-b. GSEA-based enrichment map representations based on ranked mRNA showing overlapping gene sets for the discovery and non-overlapping RNA sequencing (ICGC, **a.**) and microarray (Kool et al., **b.**) validation cohorts (p-value <0.001; FDR <0.05). Nodes (circles) representing enriched pathways identified in Group 1 are blue and Group 2 are orange. Edges connect pathways/nodes that share at least half of the terms defining them. Nodes grouped according to functional families are indicated on each network.

Supplementary Figure S6. Gene Set Enrichment Analysis overlap of discovery and validation mass spectrometry cohort. GSEA-based enrichment map representations based on ranked proteins showing overlapping gene sets for the discovery and non-overlapping LGG proteomic validation (CPTAC) cohort (p-value <0.001; FDR <0.05). Nodes (circles) representing enriched pathways identified in Group 1 are blue and Group 2 are orange. Edges connect pathways/nodes that share at least half of the terms defining them. Nodes grouped according to functional families are indicated on each network.

Supplementary Figure S8. Group 1 and Group 2 have different stromal/immune content based on ESTIMATE analysis. a-c. Box plots showing stromal (left panels), immune (center panels) or ESTIMATE (right panels) scores for discovery (**a.**), non-overlapping RNA sequencing (ICGC, **b.**) and microarray (Kool, **c.**) validation cohorts.

Supplementary Figure S9. CIBERSORT of scRNA based cell type signatures. Tumor, microglia, T cell and macrophage cell types were de-convoluted using an scRNA-based cell type signature from Reitman *et al*. using CiberSORT for discovery (**a.**), non-overlapping RNA sequencing (ICGC, **b.**) and microarray (Kool et al., **c.**) validation cohorts.

Supplementary Figure S10. Multiplex immune fluorescence analysis of PA samples. a-e. FFPE slides were stained using anti-CD4, anti-CD8, anti-IBA-1, anti-FOXP3, anti-PD1 or dapi. Results are expressed as percent total cells for anti-CD4, anti-CD8, anti-IBA-1 (**a-c**) and percent cell population for FOXP3+/CD4+ (**d**.) and PD1+/CD8+ (**e**.) cells. Plots were analyzed using Mann-Whitney test. **f**. representative immunofluorescence image of anti-PD1 (red) and dapi (blue) left, anti-CD8 (green) and dapi staining center, and the overlap between PD1+ and CD8+ cells right.
